# Synthesis, characterization, anti-ulcer action and molecular docking evaluation of novel benzimidazole-pyrazole hybrids

**DOI:** 10.1186/s13065-017-0314-0

**Published:** 2017-09-02

**Authors:** Abida Noor, Neelum Gul Qazi, Humaira Nadeem, Arif-ullah Khan, Rehan Zafar Paracha, Fawad Ali, Adil Saeed

**Affiliations:** 1 Department of Pharmaceutical Chemistry, Riphah Institute of Pharmaceutical Sciences, Riphah International University, Islamabad, Pakistan; 2 Department of Pharmacology, Riphah Institute of Pharmaceutical Sciences, Riphah International University, Islamabad, Pakistan; 3 Research Center for Modeling and Simulation, National University of Science and Technology, Islamabad, Pakistan; 4 Department of Pharmacy, Kohat University of Science and Technology, Kohat, Pakistan

**Keywords:** Benzimidazole-pyrazole, Anti-ulcer, H^+^/K^+^ ATPase, Omeprazole, Autodock vina, Molinspiration

## Abstract

A series of six novel benzimidazole-pyrazole hybrid molecules was synthesized and characterized using elemental analysis (CHN) and spectroscopic methods (^1^HNMR, FT-IR). All the synthesized compounds were evaluated for their in vivo anti ulcerogenic activity using Albino rats (weighing 180–220 g). The interactions between the compounds and active site residues of H^+^/K^+^ ATPase were investigated by molecular docking studies using autodock vina 4.0. SCH28080 was used to validate the docking results. Also the drug likeliness of these compounds was predicted using Molinspiration server in light of Lipinski’s rule of five. All the six synthesized compounds exhibited higher anti-ulcer activity as compared to omeprazole. These novel hybrid compounds showed comparable anti-ulcer potential of 72–83% at dose level of 500 µg/kg, whereas omeprazole showed 83% anti-ulcer activity at dose level of 30 mg/kg. The results clearly indicate that these novel benzimidazole-pyrazole hybrids can present a new class of potential anti ulcer agents and can serve as new anti-ulcer drugs after further investigation.

**Graphical abstract:**
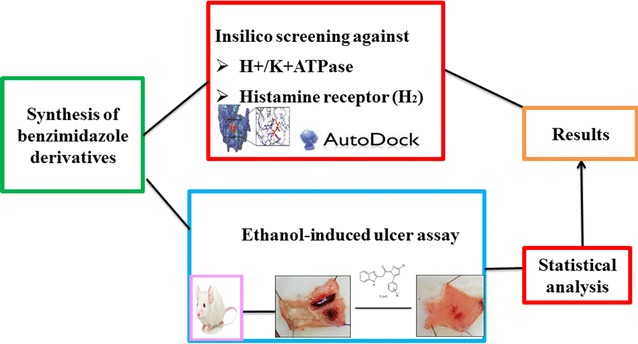
An overveiw of synthesis, in silico and in vivo antiulcer screening of benzimidazole pyrazole hybrids

An overveiw of synthesis, in silico and in vivo antiulcer screening of benzimidazole pyrazole hybrids

## Background

Peptic ulcer disease is one of the ailments that influence numerous people around the globe particularly in the developing world [1]. About 10% of the world population is affected. As a consequence of peptic ulcer about 15,000 deaths occurs annually [2]. Certain aggressive and protective factors affect the acid release in gastrointestinal tract. Any imbalance in these factors may disrupt the mucosal protection and expose gastrointestinal lining to gastric acid leading to the lesions called ulcers [3]. Various medications including proton pump inhibitors and H_2_ receptor antagonist are available for the treatment of gastric ulcers, however clinical assessment of these medications have demonstrated side effects, incidence of relapses and drug interactions [4] thus, there is need to identify more effective and safe anti-ulcer agent. The rapidly growing research in this field suggests that, with remedial and nutritional advances, gastric ulcer may become preventable within the next decade. This can be done by strengthening the defense mechanisms of the gastric mucosa and, in parallel, limiting the factors resulting in gastric ulceration. The present study focuses on the development of drugs which can reduce these damaging factors, thus preventing the ulcer formation.

With the discovery of H^+^/K^+^ ATPase as the primary gastric proton pump, inhibition of H^+^/K^+^ ATPase as a means of controlling gastric pH has gained extensive interest in recent years with the discovery of benzimidazole sulfoxide class of anti-secretory agents. Timoprazole, as one of the first well-defined inhibitor of gastric proton pump [5] which was followed by more potent picoprazole and omeprazole [6]. Synthetic benzimidazole derivatives play a major role in various pathological complications due to their high biological activity and wide range of clinical uses. The benzimidazole ring system is present in numerous anti-inflammatory [7] anti-viral [8] anti-cancer [9] and anti-microbial agents [10]. The anti-ulcer activity of sulfinyl containing benzimidazole is proved [11]. Synthetic substituted 2-mercaptobenzimidazole derivatives have been previously reported as anti-ulcer [12].

The special structural features and antiulcer potential of mercaptobenzimidazole derivatives encouraged us to synthesize some pyrazole and mercaptobenzimidazole hybrids and screen them for their anti-ulcer activity. Docking studies of the synthesized compounds were carried out against H^+^/K^+^ ATPase.

## Materials and methods

### Chemistry

All chemicals were purchased from common commercial suppliers and used without further purification. Melting points (mp) were determined on a Gallenkamp melting point apparatus and were uncorrected. The IR spectra were recorded on Thermo scientific NICOLET IS10 spectrophotometer. All ^1^H NMR and ^13^C NMR spectra were recorded on Bruker AM-300 spectrophotometer at 300 and 100 MHz respectively, in DMSO as a solvent and TMS as an internal standard at Quaid-e-Azam University, Islamabad.

#### General procedure for the preparation of compounds Synthesis of 2-mercapto benzimidazole (1)

Compound 1 was prepared according to the reported procedure [13].

#### Synthesis of ethyl 2-(benzimidazolylthio) acetate (2)

An equimolar solution of 2-mercapto benzimidazole (1) (1.50 g, 0.01 mol) and ethylchloroacetate (1.22 mL, 0.01 mol) in dry acetone (4 mL) in presence of anhydrous K_2_CO_3_ (1 g) was refluxed on a water bath for 6 h. The solvent was removed by vacuum distillation and the residue was recrystallized from chloroform to furnish compound 2 (1.055 g, 70%). m. p.: 60 64 °C; IR (cm^−1^) 3042 (SP^2^ CH), 1722 (C=O of ester), 1684 (C=N), 1320 and 1234 (C–O–C), ^1^H-NMR (300 MHz, DMSO-*d*_*6*_) ppm: 1.40 (t, 3H, J = 7 Hz, CH_3_), 4.08 (q, 2H, J = 6.75 Hz, CH_2_), 4.68 (s, 2H, S–CH_2_), 6.93–7.78 (m, 4H, Ar–H), 11.2 (s, 1H, NH). Anal. calcd. For C_11_H_12_N_2_O_2_S: C, 55.93; H, 5.10; N, 11.86. Found: C, 55.83; H, 5.04; N, 11.75.

#### Synthesis of [(2-benzimidazolylthio)-acetyl]-hydrazine (3)

Compound 2 (2.36 g, 0.01 mol) and hydrazine hydrate (0.9 mL, 0.02 mol) in ethanol (20 mL) were refluxed for about 5 h on oil bath. After cooling, the resulting solid was filtered, dried and recrystallized from ethanol to obtain compound 3 (1.77 g, 75%). m. p.: 190–193 °C; IR (cm^−1^): 3311, 3369 (NHNH_2_), 1680 (C=O); ^1^H-NMR (300 MHz, DMSO-*d*_*6*_) ppm: 4.02 (s, 2H, NH2), 4.45 (s, 2H, S–CH_2_), 7.05–7.95 (m, 4H, Ar–H), 10.55 (s, 1H, NH). Anal. calcd. for C_9_H_10_N_4_OS: C, 48.64; H, 4.80; N, 25.22. Found: C, 47.99; H, 4.69; N, 25.20.

#### General procedure for the synthesis of benzimidazole-pyrazole hybrids

Equimolar quantities of compound 3 (0.5 g, 0.001 mol) and respective chalcones (0.001 mol) were dissolved in ethanol (50 mL) containing 2–3 mL of glacial acetic acid. A few drops of hydrochloric acid were added as catalyst and the reaction mixture was refluxed for 16–17 h until the completion of reaction. After cooling, the resulting solution was added to ice cold water and resultant precipitates were collected by filtration.

##### 2-(1H-benzimidazol-2-ylsulfanyl)-1-[5-(2-hydroxyphenyl)-3-phenyl-1H-pyrazol-1-yl]ethanone (5a)

Yield 65%, m. p. 190 °C, IR (cm^−1^). 3340 (OH), 1697 (C=O), 1537 (C=C), 1617 (C=N), ^1^HNMR (300 MHz, DMSO-*d*_*6*_) ppm: 11.12 (s, 1H, OH), 9.02 (s, 1H, NH), 7.39–8.28 (m, 13H, Ar–H), 6.97 (pyrazole H), 3.34 (s, 2H, S–CH_2_). ^13^C NMR (100 MHz, DMSO-*d*_*6*_) ppm: 157.55, 153.00, 149.35, 149.35, 145.89, 142.35, 139.74, 133.35, 131.85, 128.92, 128.70, 128.70, 125.76, 125.45, 125.45, 122.53, 122.45, 121.82, 119.87, 118.01, 115.45, 107.23, 107.18, 32.10. Anal. calcd. for C_24_H_18_N_4_O_2_S: C, 67.60; H, 4.22; N, 13.14. Found: C, 67.54; H, 4.20; N, 13.10.

##### 2-(1H-benzimidazol-2-ylsulfanyl)-1-[3,5-bis(2-hydroxyphenyl)-1H-pyrazol-1-yl]ethanone (5b)

Yield 67%, m. p. 185 °C, IR (cm^−1^). 2738 (OH), 1698 (C=O), 1642 (C=N), 1540 (C=C), ^1^HNMR (300 MHz, DMSO-*d*_*6*_) ppm: 11.12 (s, 1H, OH), 9.00 (s, 1H, NH), 6.95–8.29 (m, 12H, Ar–H), 7.76 (pyrazol H), 3.35 (s, 2H, S–CH_2_). ^13^C NMR (100 MHz, DMSO-*d*_*6*_) ppm: 157.45, 156.67, 153.00, 149.30, 147.35, 145.89, 142.35, 139.74, 131.85, 131.85, 128.09, 125.76, 122.53, 122.45, 121.82, 120.51, 119.87, 118.68, 118.01, 117.19, 115.45, 107.23, 107.18, 32.61. Anal. calcd. for C_24_H_18_N_4_O_3_S: C, 65.15; H, 4.07; N, 12.66. Found: C, 65.13; H, 4.03; N, 12.62.

##### 2-(1H-benzimidazol-2-ylsulfanyl)-1-[5-(2-hydroxyphenyl)-3-(3-hydroxy-4-methoxyphenyl) -1H-pyrazol-1-yl]ethanone (5c)

Yield 59%, m. p. 195 °C, IR (cm^−1^): 3121 (OH), 1695 (C=O), 1632 (C=N), 1535 (C=C), ^1^HNMR (300 MHz, DMSO-*d*_*6*_) ppm: 14.18 (s, 1H, OH), 7.33–8.29 (m, 11H, Ar–H), 7.56 (pyrazol H), 4.01 (s, 3H, OCH_3_), 3.33 (s, 2H, S–CH_2_). ^13^C NMR (100 MHz, DMSO-*d*_*6*_) ppm; 158.43, 156.67, 149.35, 148.16, 147.35, 145.89, 145.85, 142.35, 139.74, 131.85, 131.85, 129.05, 128.09, 122.53, 122.45, 120.51, 119.87, 118.68, 117.19, 112.24, 111.02, 107.23, 107.18, 56.15, 32.73. Anal. calcd. for C_25_H_20_N_4_O_4_S: C, 63.55; H, 4.23; N, 11.86. Found: C, 63.51; H, 4.22; N, 11.85.

##### 2-(1H-benzimidazol-2-ylsulfanyl)-1-[5-(4-hydroxyphenyaminol)-3-(2-hydroxyphenyl)-1H-pyrazol-1-yl]ethanone (5d)

Yield 61%, m. p. 200 °C, IR (cm^−1^)0.3319 (OH), 1681 (C=O), 1616 (C=N), 1485 (C=C), ^1^HNMR (300 MHz, DMSO-*d*_*6*_) ppm: 11.13 (s, 1H, OH), 9.01 (s, 1H, NH), 6.95–7.71 (m, 12H, Ar–H), 6.96 (pyrazol H), 3.34 (s, 2H, S–CH_2_). ^13^C NMR (100 MHz, DMSO-*d*_*6*_) ppm: 156.29, 154.12, 153.00,149.35, 149.32, 145.89,142.35,139.74, 138.75, 131.85, 125.76,122.53, 122.45,121.82, 119.99, 119.99, 119.87, 118.01, 115.45, 115.40, 115.40, 107.23,94.57, 32.10. Anal. calcd. for C_24_H_19_N_5_O_3_S: C, 63.01; H, 4.15; N, 15.31. Found: C, 63.02; H, 4.12; N, 15.29.

##### 2-(1H-benzimidazol-2-ylsulfanyl)-1-(3,5-diphenyl-1H-pyrazol-1-yl)ethanone (5e)

Yield 58%, m. p. 170 °C, IR (cm^−1^): 3056 (NH), 3217 (OH), 1683 (C=O), 1545 (C=N), 1446 (C=C), ^1^HNMR (300 MHz, DMSO-*d*_*6*_) ppm: 11.12 (s, 1H, OH), 9.03 (s, 1H, NH), 6.51–7.12 (m, 14H, Ar–H), 6.78 (pyrazol H), 3.32 (s, 2H, S–CH_2_). ^13^C NMR (100 MHz, DMSO-*d*_*6*_) ppm: 157.55, 149.35, 149.35, 145.89, 142.35, 139.74, 133.35, 130.65, 128.92, 128.92, 128.70, 128.70, 128.66, 128.66, 128.35, 128.35, 125.45, 125.45, 122.53, 122.45, 119.87, 107.23, 107.18, 32.10. Anal. calcd. for C_24_H_18_N_4_OS: C, 70.24; H, 4.39; N, 13.65. Found: C, 70.21; H, 4.36; N, 13.62.

##### 2-(1H-benzimidazol-2-ylsulfanyl)-1-[5-(3-hydroxy-4-methoxyphenyl)-3-phenyl-1H-pyrazol-1-yl]ethanone (5f)

Yield 59%, m. p. 195 °C, IR (cm^−1^): 3121 (OH), 1695 (C=O), 1632 (C=N), 1535 (C=C), ^1^HNMR (300 MHz, DMSO-*d*_*6*_) ppm: 14.01 (s, 1H, OH), 7.23–8.09 (m, 13H, Ar–H), 7.06 (pyrazol H), 4.01 (s, 3H, OCH_3_), 3.36 (s, 2H, S–CH_2_–CO). ^13^C NMR (100 MHz, DMSO-*d*_*6*_) ppm: 157.50, 149.35, 149.30, 148.16, 145.89, 145.85, 142.35, 139.74, 133.30, 131.85, 129.05, 128.92, 128.70, 128.70, 125.40, 125.40, 122.53, 122.45, 119.87, 112.24, 111.02107.23, 107.18, 56.15, 32.10. Anal. calcd. for C_25_H_20_N_4_O_3_S: C, 65.78; H, 4.38; N, 12.28. Found: C, 65.75; H, 4.37; N, 12.27.

### Pharmacological assay

#### Animals

Albino rats (weighing 180–220 g) were housed at the animal house of the Riphah Institute of Pharmaceutical Sciences under controlled environment (23–25 °C). Animals were kept in plastic cages with sawdust (changed at every 48 h) and were fasted for 24 h before starting the experiment. Animals were provided with tap water ad libitum and standard pellet diet. Experiments performed complied with rules of Institute of Laboratory Animal Resources, Commission on Life Sciences University, National Research Council (1996) and were approved by Ethical Committee of Riphah Institute of Pharmaceutical Sciences, Riphah International University.

#### Anti-ulcerogenic activity

Albino rats (180–220 g) of either sex were divided into different groups (n = 5). Animals were fasted for 24 h before the study, but had free access to water. Animals in the control group received only normal saline (10 mL/kg). Compound 5a at doses of 100 and 500 µg/kg, (p. o.) was given to the animals in the treatment group. Same procedure was repeated for Compund 5b, 5c, 5d, 5e and 5f. Omeprazole (30 mg/kg) was used as a standard. The rats were sacrificed 1 h later and the stomach removed and observed for ulcers in the glandular region [3]. The surface area of each lesion was measured and scored by method with described by Tan et al. [14] with some modifications. The ulcer index for each rat was taken as the mean ulcer score (0: no ulcer; 1: US ≤ 0.5 mm^2^; 2: 0.5 < US ≤ 2.5 mm^2^; 3: 2.5 mm^2^ < US ≤ 5 mm^2^; 4: 5 mm^2^ < US ≤ 10 mm^2^; 5: 10 mm^2^ < US ≤ 15 mm^2^; 6: 15 mm^2^ < US ≤ 20 mm^2^; 7: 20 mm^2^ < US ≤ 25 mm^2^; 8: 25 mm^2^ < US ≤ 30 mm^2^; 9: 30 mm^2^ < US ≤ 35 mm^2^; 10: US > 35 mm^2^). The sum of the length (mm) of all the lesions for each stomach was used as the ulcer index (UI). The percentage of inhibition (% I) was calculated using the following formula:

$$\% {\text{I}} = \, \left( {{\text{USc}}\,{-}\,{\text{USt}}} \right) \, \times \, 100/{\text{USc}}$$

where USc = ulcer surface area of control and USt = ulcer surface area of test animal [14].

### Docking studies

#### Drug likeliness evaluation

Molinspiration server was used to predict number of rotatable bonds, hydrogen bond acceptors and hydrogen bond donors. These parameters help in evaluation of drug likeliness in light of Lipinski’s rule of five [15]. According to this rule, for any compound to be a good drug candidate, it should have Molecular weight (MW) less than 500 Da, H-bond donors (HBD) less than 5, H-bond acceptors (HBA) less than 10, LogP value less or equal to 5 and total rotatable bonds less than 10.

#### Molecular docking

The 3D structures of SCH28080, ligands (5a–5f), omeprazole, it’s sulfenic acid and sulfenamide derivatives were drawn using DS Visualizer v16.1.0.15350 and saved in protein data bank (PDB) format. Polar hydrogens and charges were added by Autodock tools-1.5.6.

The three dimensional (3D) structure of Pig Gastric H^+^/K^+^ ATPase (PDB code 2XZB) was retrieved from protein data bank [16]. The protein structure downloaded from protein data bank was used without any modification.

SCH28080 was used to validate the docking results because the PDB structure (2XZB) used in the docking analysis was obtained from enzyme crystallized along with it and its binding site was evaluated according to bound SCH28080 in the literature [16]. SCH28080 is a well known competitive inhibitor of gastric H^+^/K^+^ ATPase having comparable activity as of omeprazole [17]. Available literature indicate that omeprazole gets converted to its sulfenic acid and sulfenamide derivative in acidic environment and these forms bind to the Cys 813 sulfhydral group by making a covalent disulfide linkage [18, 19]. Due to this reason sulfenic acid and sulfenamide derivatives of omeprazole were also docked along with synthesized compounds.

Molecular docking was carried out by help of Pyrx 0.8 and selecting Autodock vina as docking software [20]. Vina search space coordinates were set as x = 29.161, y = 34.533 and z = −70.686. Dimensions of search space were set as x = 29.279, y = 20.040 and z = 27.678. Exhaustiveness was set at 100. All docked poses were saved in PDB format for further analysis on PyMOL Version 1.7.4.5 Edu and DS Visualizer v16.1.0.15350 [21].

### Acute toxicity test

The test was performed using increasing doses (10, 30 and 100 mg/kg) of the test compounds, given orally in 10 mL/kg volume to rats. The animals were allowed food ad libitum and kept under observation for mortality in 24 h [22].

### Statistical analysis

Data expressed are mean ± standard error of mean (SEM, n = number of experiment). The statistical parameter applied is one-way analysis of variance with post hoc Tukey test, *P* < 0.05 noted as significantly different.

## Results and discussion

### Chemistry

**Fig. 1:**
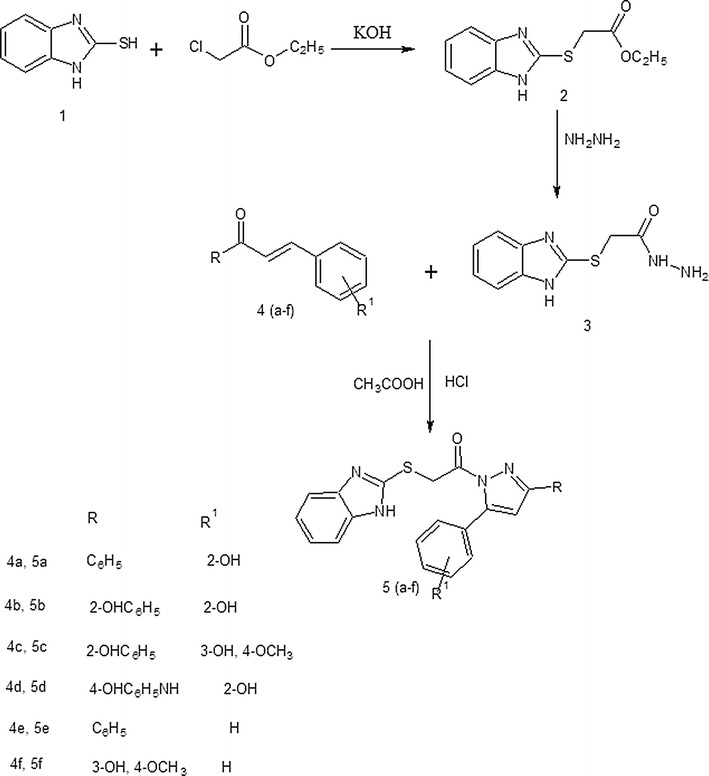
Synthesis of Mercatobenzimidazole-Pyrazole-hybrid derivatives

As shown in scheme, the benzimidazole-Pyrazole 5 (a–f) hybrids were prepared by three step synthesis starting from 2-mercaptobenzimidazole **1**. Treatment of compound 1 with ethyl chloroacetate in the presence of KOH gave the corresponding ethyl 2-(benzimidazolylthio) acetate **2**, which was further condensed with hydrazine hydrate to afford the hydrazide 3 in 75% yield. The hydrazide was further reacted with six different chalcones **4a**–**f** in the presence of acetic acid and hydrochloric acid and respective pyrazole derivatives **5a**–**f** were obtained in 60–70% yield (Fig. 1). All the compounds were purified by recrystallization in suitable solvents. The structures of compounds **5a**–**f** were confirmed by IR, NMR spectroscopic data and mass analysis. The IR spectra showed the carbonyl peak for amide and OH, NH stretching vibrations. The ^1^H NMR data of all the final compounds **5a**–**f** showed a singlet at δ 6.95–7.76 ppm which was characteristic of an aromatic proton of pyrazole ring. Methylene protons attached to sulfur atom were observed at δ 3.34 ppm, while all other aromatic protons resonated in the region δ 6.95–8.29 ppm. In compound **5d** and **5f**, a singlet of methoxy protons resonated at δ 4.01 ppm. Singlet of NH and OH protons were observed downfield in all the compounds.

### Anti-ulcerogenic assay

**Fig. 2:**
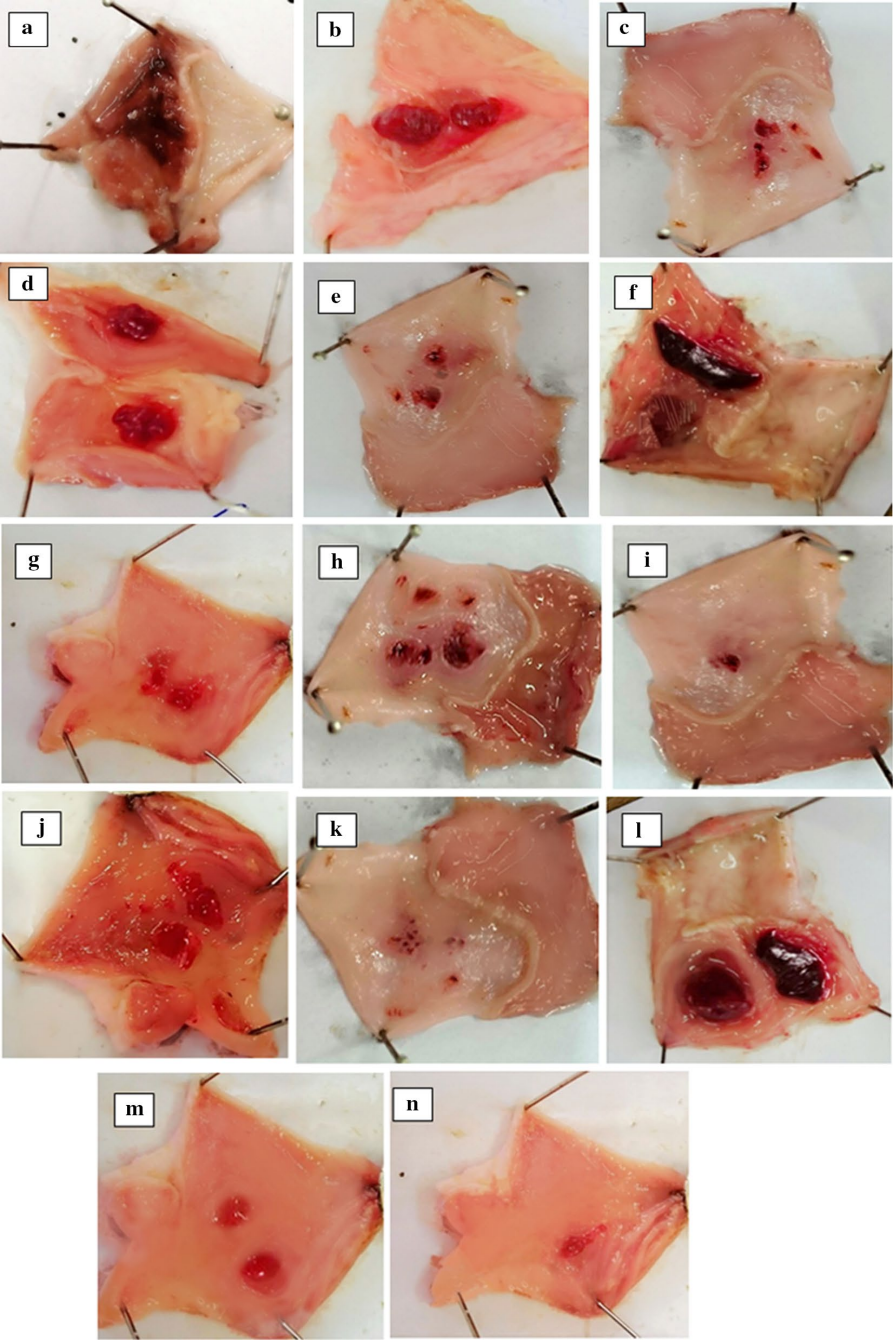
Gross appearance of gastric mucosa in rats: **a** pre-treated with saline, 10 mL/kg (ulcer control). Severe injuries are seen, as absolute ethanol (1 mL/100 g) produced excessive visible hemorrhagic necrosis in gastric mucosa **b** and **c** pre-treated with compound 5a at doses of 100 and 500 µg/kg, **d** and **e** pretreated with compound 5b at doses of 100 and 500 µg/kg, **f** and **g** pre-treated with compound 5c at doses of 100 and 500 µg/kg, **h** and **i** pre-treated with compound 5d at doses of 100 and 500 µg/kg, **j** and **k** pre-treated with compound 5e at doses of 100 and 500 µg/kg, **l** and **m** pre-treated with compound 5f at doses of 100 and 500 µg/kg and **n** pre-treated with omeprazole 30 mg/kg. The injuries reduce with increase of compounds doses and omeprazole, compared to ulcer control

The anti-ulcer activity of all the benzimidazole-pyrazole hybrids **5a**–**f** was tested in vivo by ethanol-induced gastric ulcer model in rats. Rats received test compounds in two different doses; 100 and 500 µg/kg orally. Omeprazole (30 mg/kg) was used as a standard. The rats were sacrificed 1 h later and the stomach removed and observed for ulcers in the glandular region [3]. The surface area of each lesion was measured and scored by method [14]. All compounds (5a–f) exhibited anti-ulcer effect. Figure 2 show the gastric mucosa of rats. Compound 5a at 100 and 500 µg/kg caused 35.4 and 78.5% (*P* < 0.001 versus saline group) inhibition respectively, compound 5b at 100 and 500 µg/kg caused 30.2 and 74.7% inhibition respectively (*P* < 0.001 versus saline group), compound 5c exhibited 29.4 and 70.3% inhibition of ulcer formation at 100 and 500 µg/kg respectively (*P* < 0.001 versus saline group), compound 5d at 100 and 500 µg/kg showed 38.1 and 83.1% inhibition respectively (*P* < 0.001 versus saline group), compound 5e exhibited 36.2 and 79% inhibition at 100 and 500 µg/kg respectively (*P* < 0.001 versus saline group), compound 5f at 100 and 500 µg/kg showed 24.8 and 72.1% inhibition respectively (*P* < 0.001 versus saline group) and omeprazole (30 mg/kg) exhibited 83.1% inhibitory effect (Table 1). The results clearly indicated the greater anti-ulcer potential of our synthesized compounds as compared to omeprazole, the currently used anti-ulcer drug. All the compounds 5a–f showed higher antiulcer activity at higher dose level of 500 µg/kg. It was observed that substitution pattern of both the aromatic rings attached to pyrazole ring effect the anti-ulcer activity. The highest activity was shown by compound 5d i.e. 83.1% at 500 µg/kg dose. All the compounds were docked against H^+^/K^+^ ATPase to assess the binding affinities and compared with Omeprazole which is a known mercapto benzimidazole derivative and H^+^/K^+^ ATPase inhibitor. As suggested by computational studies, all compounds bind with H^+^/K^+^ ATPase having good binding affinities. 5d has maximum binding affinity of −9.8 kcal/mole due to maximum number of hydrogen bonding interactions with the target. Compound 5e and 5a also exhibited good binding affinities of −9.4 and −9.5 kcal/mole, whereas in case of compound 5c and 5f binding affinity is less which can be attributed to the presence of 3-OH group which leads to less binding affinity as compared to 4-OH, also the replacement of 4-OH by 4-OCH3 group in 5a and 5f resulted in disappearance of hydrogen bonding and hence decreased the binding energy. As evident from results the OH group of ring A is not involved in hydrogen bonding and therefore its absence in case of 5a increased the binding affinity, while the ortho hydroxyl group of ring B increased binding affinity as it is involved in hydrogen bonding with the target.

**Table 1:** Protective effect of compounds and omeprazole against ethanol-induced gastric ulcers in rats

Treatment	Ulcer index	% Inhibition
Saline 10 mL/kg + Ethanol (1 mL/100 g)	4.99 ± 0.007	–
Compund 5a (100 µg/kg) + Ethanol (1 mL/100 g)	3.22 ± 0.009***	35.4
Compound 5a (500 µg/kg) + Ethanol (1 mL/100 g)	1.07 ± 0.008***	78.5
Compund 5b (100 µg/kg) + Ethanol (1 mL/100 g)	3.48 ± 0.008***	30.2
Compound 5b (500 µg/kg) + Ethanol (1 mL/100 g)	1.26 ± 0.009***	74.7
Compund 5c (100 µg/kg) + Ethanol (1 mL/100 g)	3.52 ± 0.009***	29.4
Compound 5c (500 µg/k g) + Ethanol (1 mL/100 g)	1.48 ± 0.009***	70.3
Compund 5d (100 µg/kg) + Ethanol (1 mL/100 g)	3.09 ± 0.007***	38.1
Compound 5d (500 µg/kg) + Ethanol (1 mL/100 g)	0.84 ± 0.017***	83.1
Compund 5e (100 µg/kg) + Ethanol (1 mL/100 g)	3.18 ± 0.01***	36.2
Compound 5e (500 µg/kg) + Ethanol (1 mL/100 g)	1.06 ± 0.01***	79
Compund 5f (100 µg/kg) + Ethanol (1 mL/100 g)	3.75 ± 0.016***	24.8
Compound 5f (500 µg/kg) + Ethanol (1 mL/100 g)	1.39 ± 0.02***	72.1
Omeprazole (30 mg/kg) + Ethanol (1 mL/100 g)	0.84 ± 0.01***	83.1

*** *P* < 0.001 compared to control saline group, one-way analysis of variance with post hoc Tukey test, n = 5

Recently some new derivatives of mercapto benzimidazole have been reported for their strong inhibitory potential against H^+^/K^+^ATPase. These compounds include 2-[3-(2,3-dihydro-1Hpyrolo[1,2a]benzimidazolyl)sulfinyl]-5-methyl-1H benzimidazoles, 20379-4 [23] 5,7-dihydro-2{[(4methoxy-3-methyl-2-pyridyl)methyl]sulfinyl}-5,5,7,7-tetramethylindeno-[5,6d]imidazole-6-(1H)-one (Ro185364), [24], 4-(N-allyl-N-methylamino)-1-ethyl-8-[(5-fluoro-6-methoxy-2-benzimidazolyl) sulfinylmethyl]1-ethyl-1,2,3,4-tetrahydroquinoline [25] and 2-(1H-benzoimidazole-2-sulfinylmethyl)-4-dimethylaminopyrimidine-5-carboxylicacid ethyl ester [26]. Therefore, we decided to synthesize the target molecules and screen them for antiulcer activity. The proposed hypothesis was that our compounds (5a–f) being resembling with omeprazole and SCH28080 might have antiulcer potential through the inhibition of H^+^/K^+^ ATPase. To check the hypothesis docking studies were carried out and very good binding energies of our compound with the target comparable with omeprazole and SCH28080 were obtained. This suggests that H^+^/K^+^ATPase inhibition is a possible mechanism of action of these compounds, but involvement of other mechanisms cannot be ignored. Hypothesis can be validated through in vitro studies, which is part of our future plans.

**Fig. 3:**
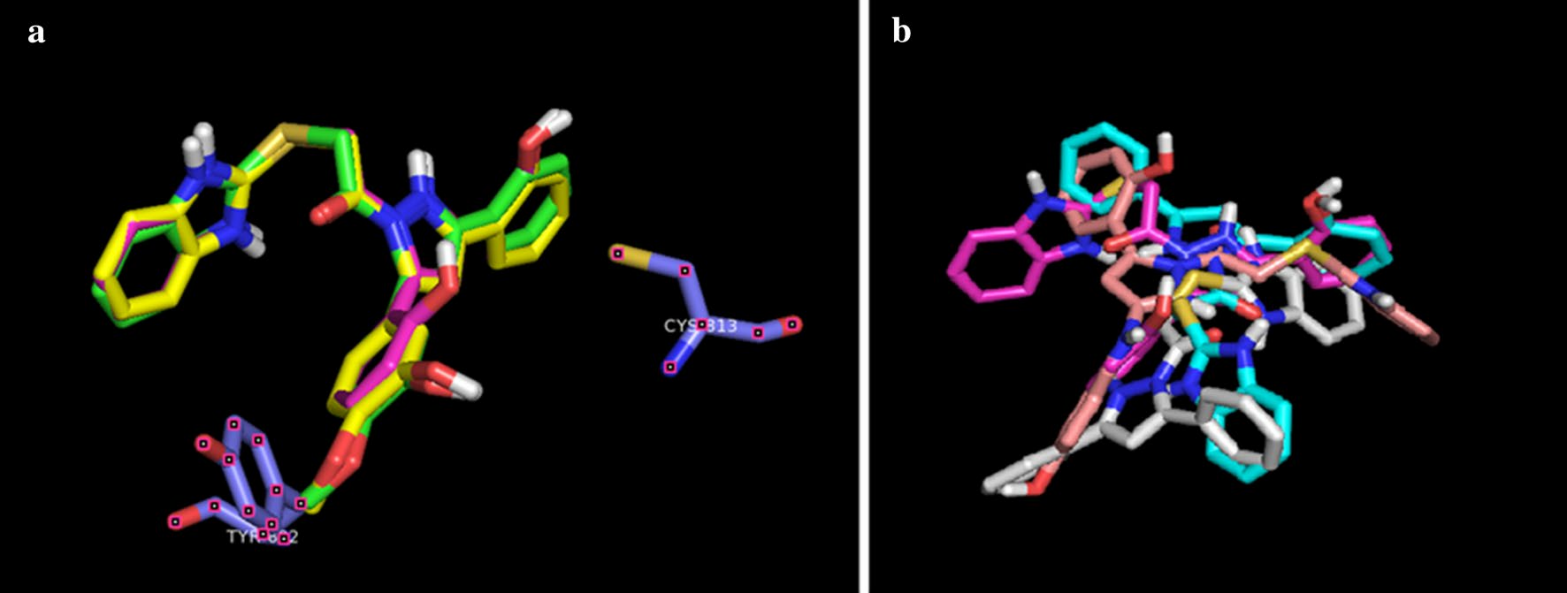
5b, 5c and 5f between Cys 813 and Tyr 802 of binding pocket (**a**) their binding poses are same and are overlapping with each other. 5a, 5b, 5d and 5e have different binding poses (**b**)

**Fig. 4:**
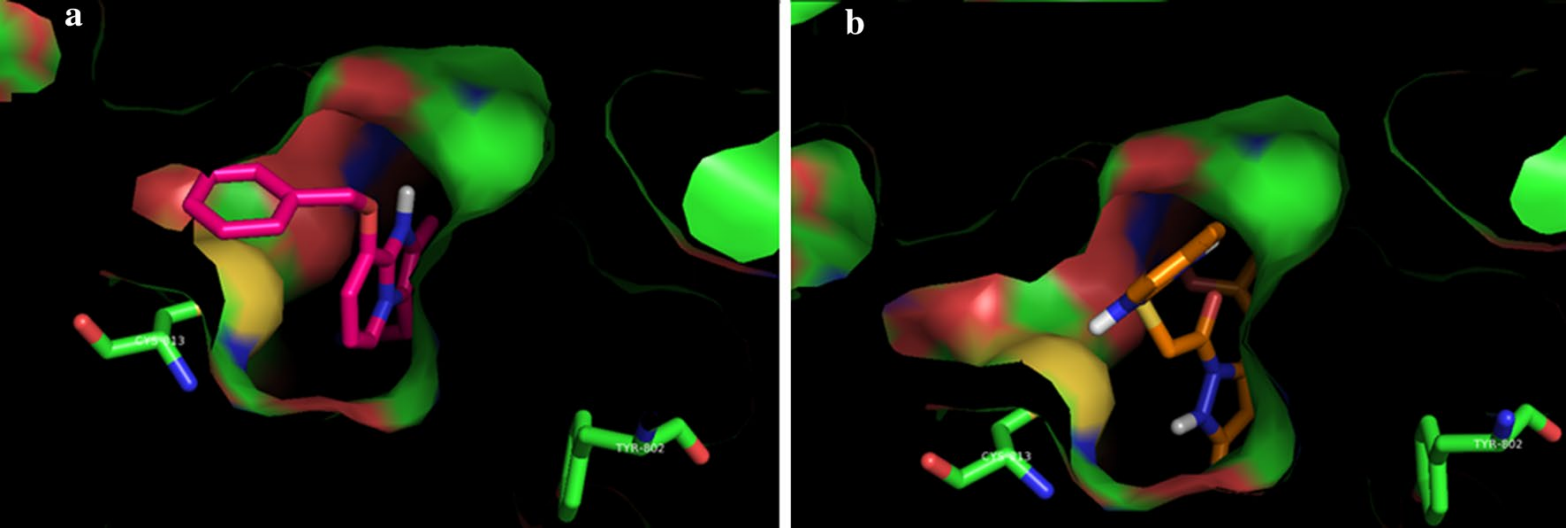
SCH28080 covering the Cys813 in the active site (**a**). Compound 5d covering the Cys 813 in the active site (**b**)

### In-silico drug likeliness assessment

Drug-likeness or drugability of molecules was assessed based on Lipinski rule of five as suggested by Christopher A. Lipinski. According to Lipinski’s ‘rule-of-five’ drugs should have a molecular weight of ≤500 Da, a logP ≤ 5, hydrogen bond donor ≤5 and hydrogen bond acceptor sites (N and O atoms) ≤10 that they have strong absorption.

Drug likeliness of our synthesized compounds is given in Table 2. It indicated that compounds 5c and 5f are fulfilling all the criteria of lipinski’s rule of five. The Predicted values of miLogP for all compounds are around 5. In general all the synthesized compounds have identical activity according to milogP predictions. Compound 5d has maximum number of hydrogen bond acceptor and hydrogen bond donor properties and highest total polar surface area of 116.06. Topological polar surface area (TPSA) was calculated for all the compounds. It should be ≤140 5b of a molecule which correlates well with the passive molecular transport through membranes.

**Table 2:** Druglikeliness

S. no	Ligand	miLog P	TPSA	Molecular weight	No. of H-bond acceptor	No. of H-bond donor	No. of violations	No. of rotatable bonds	Volume
1	5a	5.38	83.81	426.50	6	2	1	5	366.23
2	5b	5.11	104.04	442.50	7	3	1	5	374.25
3	5c	4.72	113.27	472.53	8	3	0	6	399.80
4	5d	5.34	116.06	457.51	8	4	1	6	386.65
5	5e	5.64	63.58	410.50	5	1	1	5	358.21
6	5f	4.98	93.04	456.53	7	2	0	6	391.78
7	Omeprazole	2.41	77.11	345.42	6	1	0	5	302.81

All the compounds have TPSA within the range. The drug-likeness data of our compounds suggested that these compounds can be used as drugs because all of the above mentioned descriptors for these molecules are within the range.

### Molecular docking analysis

Docking scores of first three best docked posses are given in Table 3. Docking scores predict that all the synthesized compounds (5a–5f) will show slight difference in activity with 5d having highest activity and compounds 5c and 5f having lowest activity of all. These docking results are in accordance with the results of in vivo activity.

**Table 3:** Docking scores of first three best docked posses

Ligand	Binding affinity (kcal/mol)	Mode	rmsd/ub	rmsd/lb
5a	−9.5	0	0	0
5a	−9.5	1	7.294	2.981
5a	−9.4	2	8.366	3.478
5b	−9.4	0	0	0
5b	−9.4	1	8.305	3.3
5b	−9.4	2	7.025	3.268
5c	−9.2	0	0	0
5c	−9	1	6.477	3.593
5c	−9	2	8.947	4.176
5d	−9.8	0	0	0
5d	−9.5	1	8.682	2.897
5d	−9.5	2	6.742	3.731
5e	−9.4	0	0	0
5e	−9.4	1	8.741	4.258
5e	−9.3	2	7.866	4.377
5f	−9.2	0	0	0
5f	−8.9	1	8.67	4.143
5f	−8.8	2	6.344	3.617
SCH28080	−7.7	0	0	0
SCH28080	−7.7	1	1.855	1.386
SCH28080	−7.6	2	2.239	1.821
Omeprazole	−7.7	0	0	0
Omeprazole	−7.7	1	7.939	2.578
Omeprazole	−7.6	2	2.229	1.456
SA	−7.8	0	0	0
SA	−7.2	1	3.942	2.388
SA	−7	2	10.687	8.036
SC	−7.7	0	0	0
SC	−7.4	1	7.333	2.091
SC	−7.3	2	11.516	9.22

Docking scores of synthesized compounds is also better than SCH28080, omeprazole, its sulfenic acid derivative (SC) and sulfenamide derivative (SA) predicting that synthesized compounds will be more active. This is also observed in in vivo activity test as dose for omeprazole was very higher than the dose of compounds (5a–f) suggesting that these compounds are far more potent than omeprazole.

In post docking analysis it was observed that compound 5b, 5c, and 5f are binding to the active site in almost same pose, whereas, 5a, 5d, and 5e are binding to the active site in totally different pose. According to docking score poses of compounds 5a, 5d and 5e are energetically better than the posses of compounds 5b, 5c and 5f as shown in Fig. 3.

**Fig. 5:**
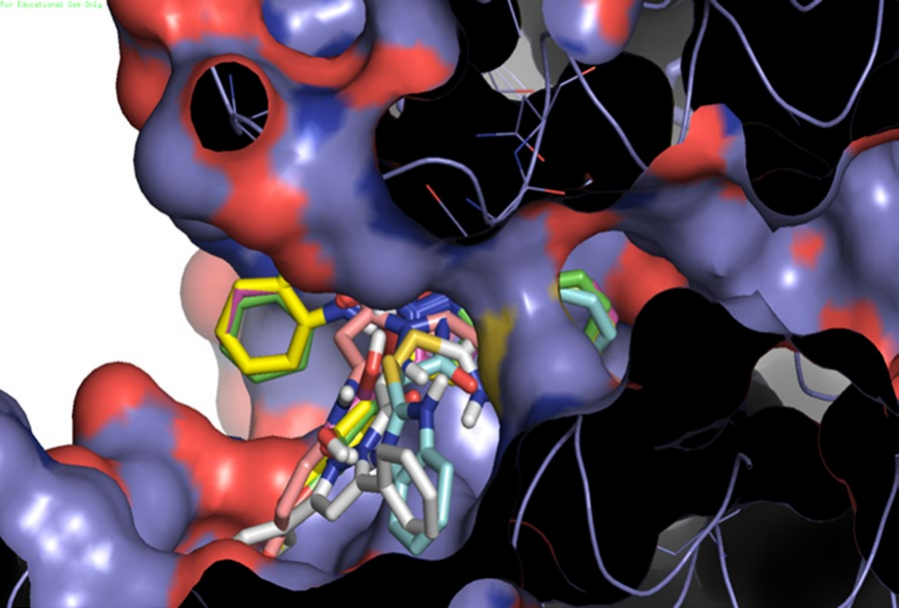
Componds 5a–5f posses in binding pocket

SCH28080 covers the Cys 813 in the binding pocket. It can be observed that compound 5d covers the Cys 813 in the binding pocket in the same way as SCH28080 as expressed in Fig. 4. In Fig. 5 all compounds (5a–f) are shown docked in the binding pocket depicting that all compounds are binding to the same site with little variation in binding pose.

**Fig. 6:**
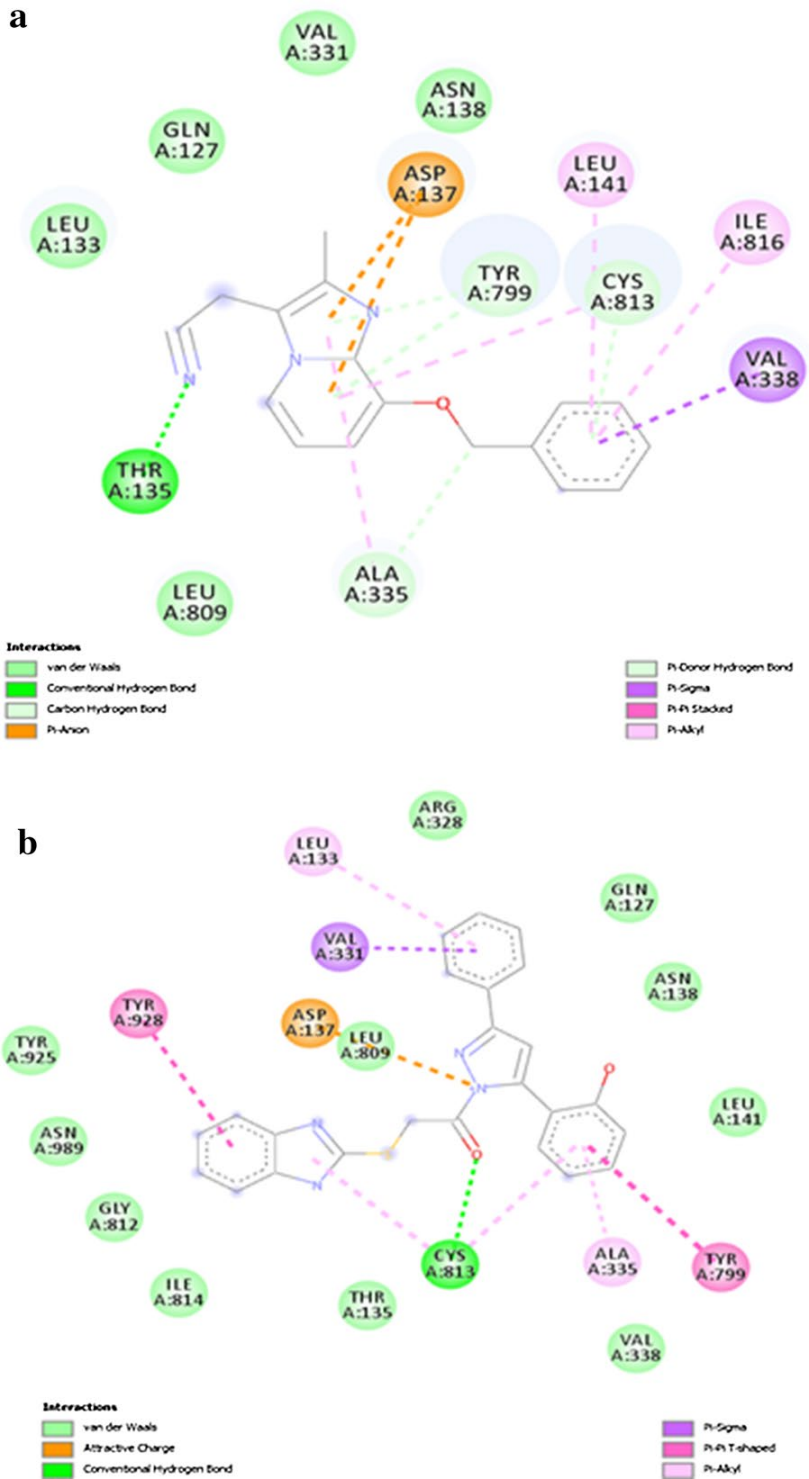
Predicted interactions of SCH28080 (**a**) and 5a (**b**) with active site

**Fig. 7:**
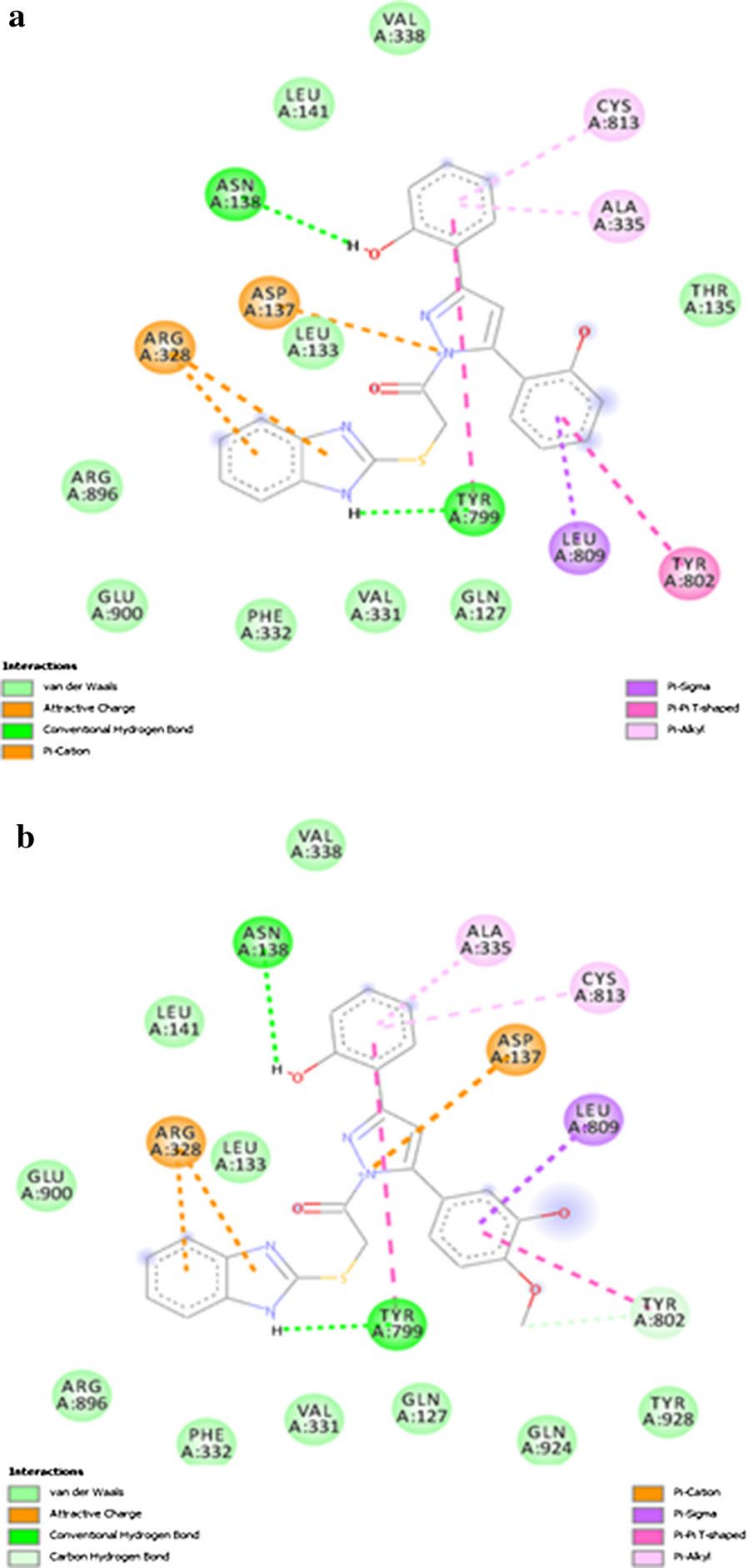
Predicted interactions of 5b (**a**) and 5c (**b**) with active site

**Fig. 8:**
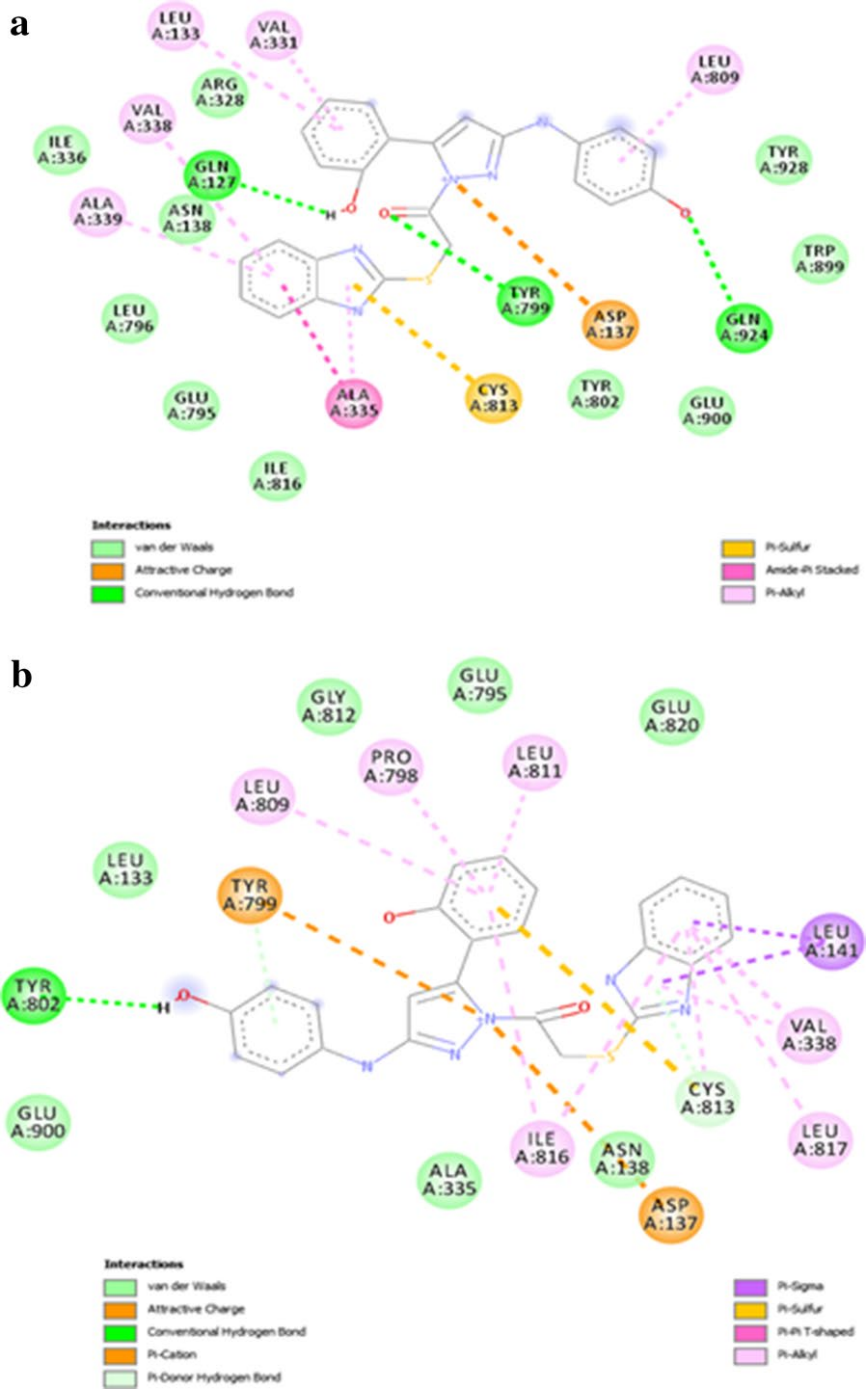
Predicted interactions of two poses of 5d (0 and 2) (a and b) with active site

**Fig. 9:**
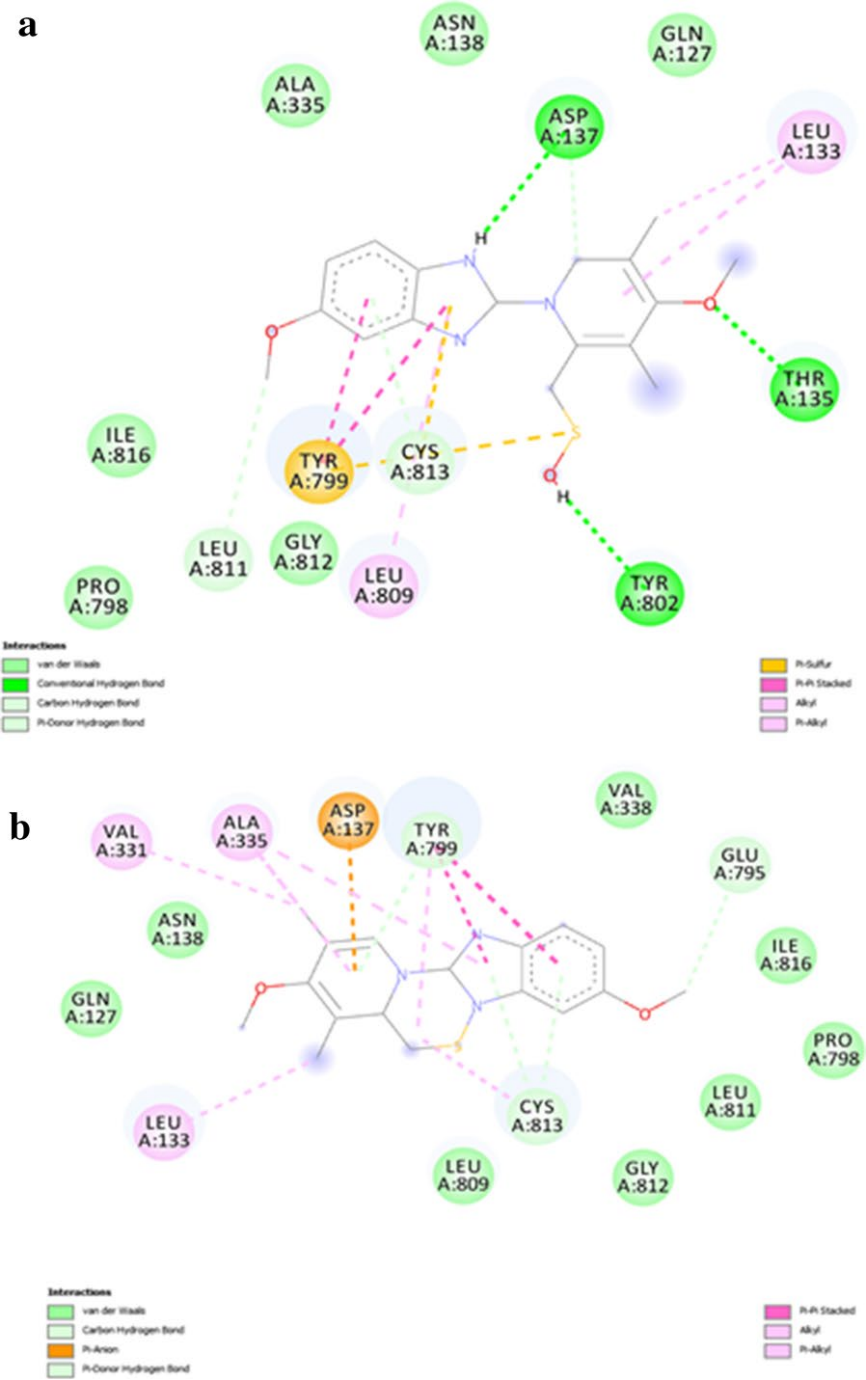
Predicted interactions of 5e (**a**) and 5f (**b**) with active site

**Fig. 10:**
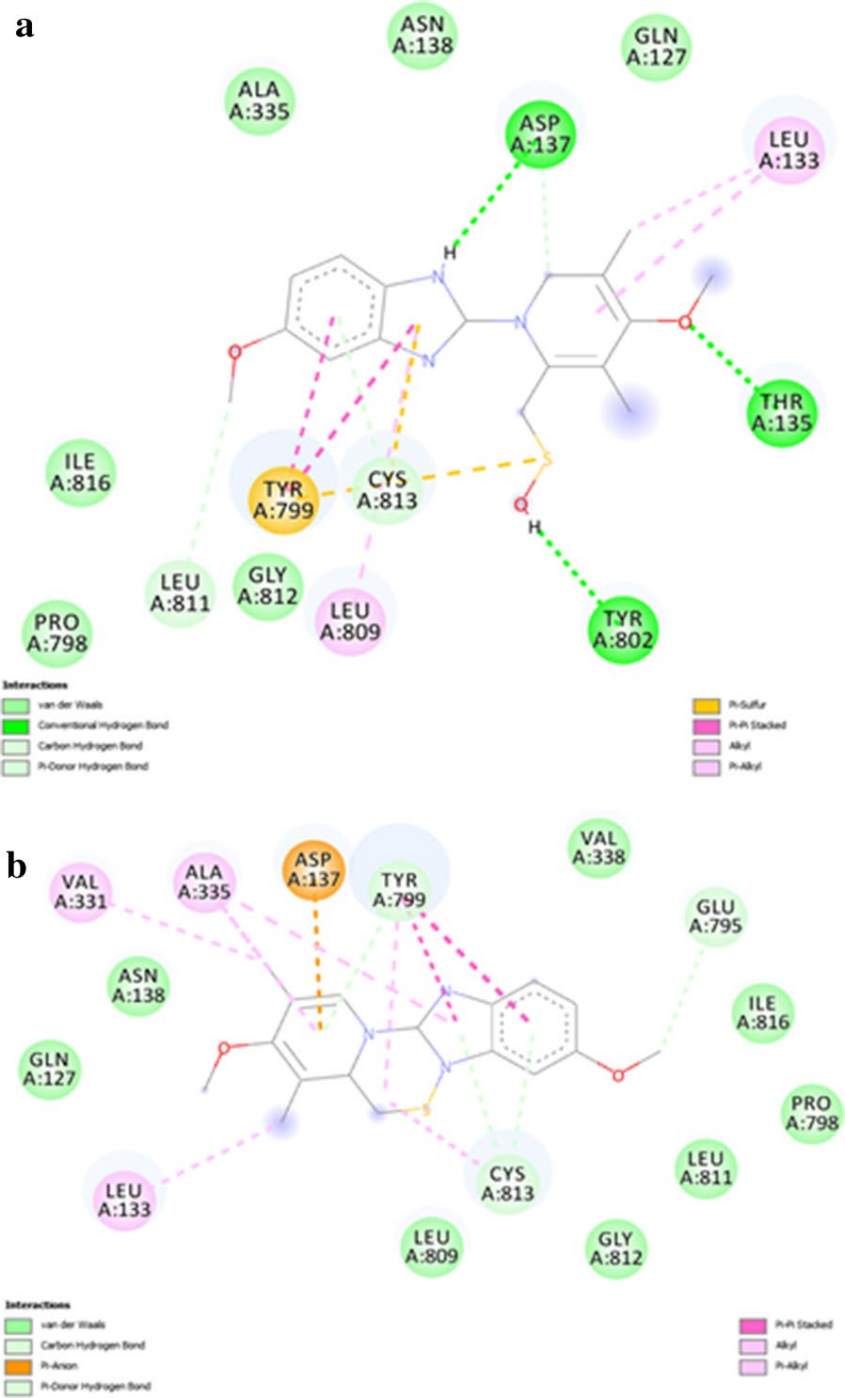
Predicted interactions of omeprazole’s sulfenic acid (**a**) and sulfenamide (**b**) derivatives with active site

All of the compounds (5a–f) are showing more interactions with the amino acid residues surrounding the active site as compared to other reference molecules. It can be seen in the Fig. 6(a) that SCh28080 has fewer interactions with active site as compared to synthesized compounds (5a–f). Highest number of interactions is predicted in case of compound 5d Fig. 7. Its two binding posses (mode 0 and 2) are given in Fig. 8a, b. Predicted interactions of omeprazole’s sulfenic acid Fig. 9 and sulphenamide derivative are shown in Fig. 10a, b. Predicted interactions of omeprazole’s sulfenamide derivate are also less than interactions of our compounds.

### Acute toxicity

The test compounds 5a, 5b, 5c, 5d and 5e did not causes any mortality up to dose of 100 mg/kg. 5f was found lethal at the dose of 100 mg/kg.

## Conclusions

Six novel benzimidazole-pyrazole hybrids were synthesized and evaluated for their anti-ulcer activity. All the compounds exhibited potent anti-ulcer activity at lower dose levels as compared to the standard, omeprazole. Further the molecular interaction of the these novel hybrids molecules with the target H^+^/K^+^ ATPase were established through docking studies. It was found that our compounds showed higher binding affinities as compare to omeprazole. This suggest that compounds (5a–f) might be acting through the same mechanism as omeprazole and other related compounds but involvement of other mechanism cannot be ignored. The study confirmed that these molecules can present a new class of lead molecules for drug discovery as H^+^/K^+^ ATPase inhibitors.
